# Visualization of the physical and functional interaction between hMYH and hRad9 by Dronpa bimolecular fluorescence complementation

**DOI:** 10.1186/1471-2199-15-17

**Published:** 2014-08-15

**Authors:** Lia Agustina, Soo-Hyun Hahm, Se Hee Han, An Hue Vy Tran, Ji Hyung Chung, Jong-Hwa Park, Jin Woo Park, Ye Sun Han

**Affiliations:** 1 Department of Advanced Technology Fusion, Konkuk University, 1 Hwayang-dong, Gwangjin-gu, Seoul 143-701, Korea; 2 Department of Applied Bioscience, College of Life Science, CHA University, Gyeonggi-do 463-836, Korea; 3 Department of Genetic Engineering and Graduate School of Biotechnology, Kyung Hee University, Yongin 446-701, Korea; 4 BioActs, DKC Corporation, 693-2 Gojan-dong, Namdong-gu, Incheon 405-820, Korea

**Keywords:** Human MYH, Human Rad9, Bimolecular fluorescence complementation, FRET, Immunofluorescence, Protein-protein interaction

## Abstract

**Background:**

Human MutY glycosylase homolog (hMYH), a component of the base excision repair pathway, is responsible for the generation of apurinic/apyrimidinic sites. Rad9-Rad1-Hus1 (9-1-1) is a heterotrimeric protein complex that plays a role in cell cycle checkpoint control and DNA repair. In humans, hMYH and 9-1-1 interact through Hus1 and to a lesser degree with Rad1 in the presence of DNA damage. In *Saccharomyces pombe*, each component of the 9-1-1 complex interacts directly with SpMYH. The glycosylase activity of hMYH is stimulated by Hus1 and the 9-1-1 complex and enhanced by DNA damage treatment. Cells respond to different stress conditions in different manners. Therefore, we investigated whether Rad9 interacted with hMYH under different stresses. Here, we identified and visualized the interaction between hRad9 and hMYH and investigated the functional consequences of this interaction.

**Results:**

Co-IP and BiFC indicates that hMYH interacts with hRad9. As shown by GST-pull down assay, this interaction is direct. Furthermore, BiFC with deletion mutants of hMYH showed that hRad9 interacts with N-terminal region of hMYH. The interaction was enhanced by hydroxyurea (HU) treatment. mRNA and protein levels of hMYH and hRad9 were increased following HU treatment. A marked increase in p-Chk1 (S345) and p-Cdk2 (T14, Y15) was observed. But this phosphorylation decreased in siMYH- or siRad9-transfected cells, and more pronounced decrease observed in co-transfected cells.

**Conclusions:**

Our data reveal that hRad9 interacts directly with N-terminal region of hMYH. This interaction is enhanced by HU treatment. Knockdown of one or both protein result in decreasing Chk1 and Cdk2 phosphorylation. Since both protein functions in the early detection of DNA damage, we suggest that this interaction occurs early in DNA damage pathway.

## Background

A major product of DNA damage, 8-oxoguanine (8-oxoG), is generated in DNA following oxidative damage by reactive oxygen species (ROS). If not repaired, this 8-oxoG results in a transversion from G:C to T:A [1]. The human MutY glycosylase homolog (hMYH) is involved in base excision repair (BER); which is initiated by recognition and removal of adenine residues from DNA. hMYH cleaves the *N*-glycosidic bond between a target base and its deoxyribose sugar. This results in an apurinic/apyrimidinic (AP) site [2]. hMYH has multiple forms that arise from multiple transcription initiation sites and alternative splicing of hMYH mRNA transcripts [3].

Rad9-Rad1-Hus1 forms a heterotrimeric protein complex called 9-1-1. The structure of this complex is similar to that of proliferating cell nuclear antigen (PCNA), a replication clamp [4]. Previous studies have shown that Rad17 recruits the 9-1-1 complex to DNA damage sites and the Rad17-Rfc2-5 complex loads the 9-1-1 complex onto primed DNA [5]. The 9-1-1 complex has several functions, such as DNA repair, cell cycle checkpoint control, BER, homologous recombination, mismatch repair, apoptosis, and 3′–5′ exonuclease activity [6-9]. The complex also interacts with and/or stimulates components of the BER pathway, including polymerase β (Polβ), flap endonuclease 1 (FEN1), replication protein A (RPA), and DNA ligase 1 (Lig1) [10-13]. hRad9 is regulated by phosphorylation and by the differential interactions with different protein partners, which likely determines the multiple functions of hRad9 [6,14].

Protein-protein interactions can be studied by several methods. Bimolecular fluorescence complementation (BiFC) is based on the complementation of two non-fluorescent fragments of a fluorophore. Interaction between proteins fused to the fragments facilitates the association of the non-fluorescent fragments [15]. Dronpa is a green fluorescent protein (GFP)-like protein that photoswitches between a fluorescent “on” state and a non-fluorescent “off” state in response to light irradiation [15]. Previously, we had successfully generated a Dronpa-BiFC system to visualize the interaction between hHus1-hMYH and an intercomplex interaction between hRad1-hHus1 [16].

In *Saccharomyces pombe*, each component of the 9-1-1 complex interacts directly with SpMYH, and these interactions were increased after treatment with H_2_O_2_[12]. Furthermore, human 8-oxoguanine DNA glycosylase (hOGG1), another DNA glycosylase, can interact with each subunit of the 9-1-1 complex. The interaction between the 9-1-1 complex and hOGG1 increase hOGG1 enzyme activity [17]. However, according to previous report by Shi *et al.*, hMYH interacts with the 9-1-1 complex through hHus1 and interacts to a lesser degree with hRad1, but not with hRad9 [18]. The glycosylase activity of hMYH is stimulated by hHus1 and the 9-1-1 complex, and this interaction is enhanced by DNA damaging treatments [18]. Therefore, it would be interesting to study the different interactions between 9-1-1 and hMYH under various conditions.

In this study, we have used a Dronpa-BiFC system and shown for the first time that hRad9 interacts with the N-terminal region of hMYH. We also examined the physiological effects of the hRad9 and hMYH interaction by exposing cells to damaging agents such as HU. The mRNA levels of hMYH and hRad9 were increased after treatment with HU, but not after treatment with H_2_O_2_. Moreover, the interaction between hMYH and hRad9 was also indicated by FRET (Fluorescence resonance energy transfer), which increased after HU treatment. Cell cycle arrest, induced by p-Chk1 (S345), is evident from the increase in the inactive form of cyclin-dependent kinase 2 (Cdk2, phosphorylated at T14, Y15). Phosphorylation of Chk1 and Cdk2 decreased in hMYH and hRad9 knockdown cells; this decrease was more pronounced after HU treatment. hRad9 interacted with hMYH as a component of the 9-1-1 complex because depletion of Hus1 reduced the interaction. We hypothesize that this interaction occurs during the early stages of DNA damage repair.

## Results and discussion

### hMYH physically interacts with hRad9

**Figure 1:**
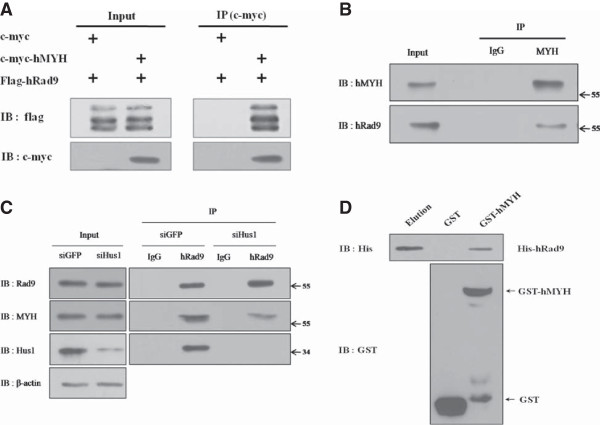
**Interaction between hMYH and hRad9. (A)** HEK293 cells were transfected with c-myc and FLAG-tagged hRad9, or c-myc-tagged hMYH and FLAG-tagged-hRad9 as indicated. Co-IP was performed with anti-c-myc antibody, and the immunoprecipitates were immunoblotted with anti-FLAG and anti-c-myc antibodies. **(B)** Lysates from untransfected HEK293 cells were used for the immunoprecipitation of hMYH with an anti-hMYH antibody. Immunoblotting was performed using anti-hMYH and anti-hRad9 antibodies. Lane 1: input sample, lanes 2 and 3: proteins immunoprecipitated with mouse IgG or anti-hMYH antibody. **(C)** HEK293 cells were transfected with siHus1 or siGFP. Total cell lysates were used for co-IP with hRad9 antibody. **(D)** Pull-down of purified His-hRad9 with GST-hMYH bound to glutathione-Sepharose beads. Lane 1: purified His-hRad9, lanes 2 and 3: pull-downs with GST and GST-hMYH, respectively.

To determine whether hRad9 interacts with hMYH, co-immunoprecipitation (co-IP) was performed using HEK293 cells transfected with c-myc and FLAG-tagged hRad9 or c-myc-tagged hMYH and FLAG-tagged hRad9. After incubation for 24 h, cell lysates were extracted and immunoprecipitated with an anti-c-myc antibody. Precipitated samples were analyzed by immunoblotting with anti-FLAG and anti-c-myc antibodies. FLAG-hRad9 was precipitated through its interaction with c-myc-hMYH (Figure 1A). In western blot analysis, hRad9 was visualized as several bands due to differences in the phosphorylation states. These results may indicate a physical interaction between hMYH and hRad9. The endogenous interaction between hRad9 and hMYH was confirmed by co-IP with anti-hMYH antibody. hRad9 immunoprecipitated with hMYH (Figure 1B), indicating that endogenous hMYH interacts with hRad9.To determine the involvement of the 9-1-1 complex in the interaction between hMYH and hRad9, Hus1 expression was knocked down using specific siRNA. In Hus1-depleted cells, the interaction between hMYH and hRad9 was decreased (Figure 1C). This result indicates that hRad9 interacts with hMYH as part of the 9-1-1 complex.

Bacterially expressed and purified His-hRad9 and GST-hMYH were used in GST pull-down assays to demonstrate the physical interaction between hMYH and hRad9. GST or GST-hMYH was immobilized on glutathione-Sepharose beads and used to pull-down purified His-hRad9. We observed a band for His-hRad9 in the pull-down assay with GST-hMYH, but not with GST alone, indicative of a direct interaction between hMYH and hRad9 (Figure 1D). Our results differ from those obtained by Shi *et al*. [18], which did not observe an interaction between hMYH and hRad9 in GST pull-down assays.

### hRad9 interacts with the N-terminal region of hMYH

**Figure 2:**
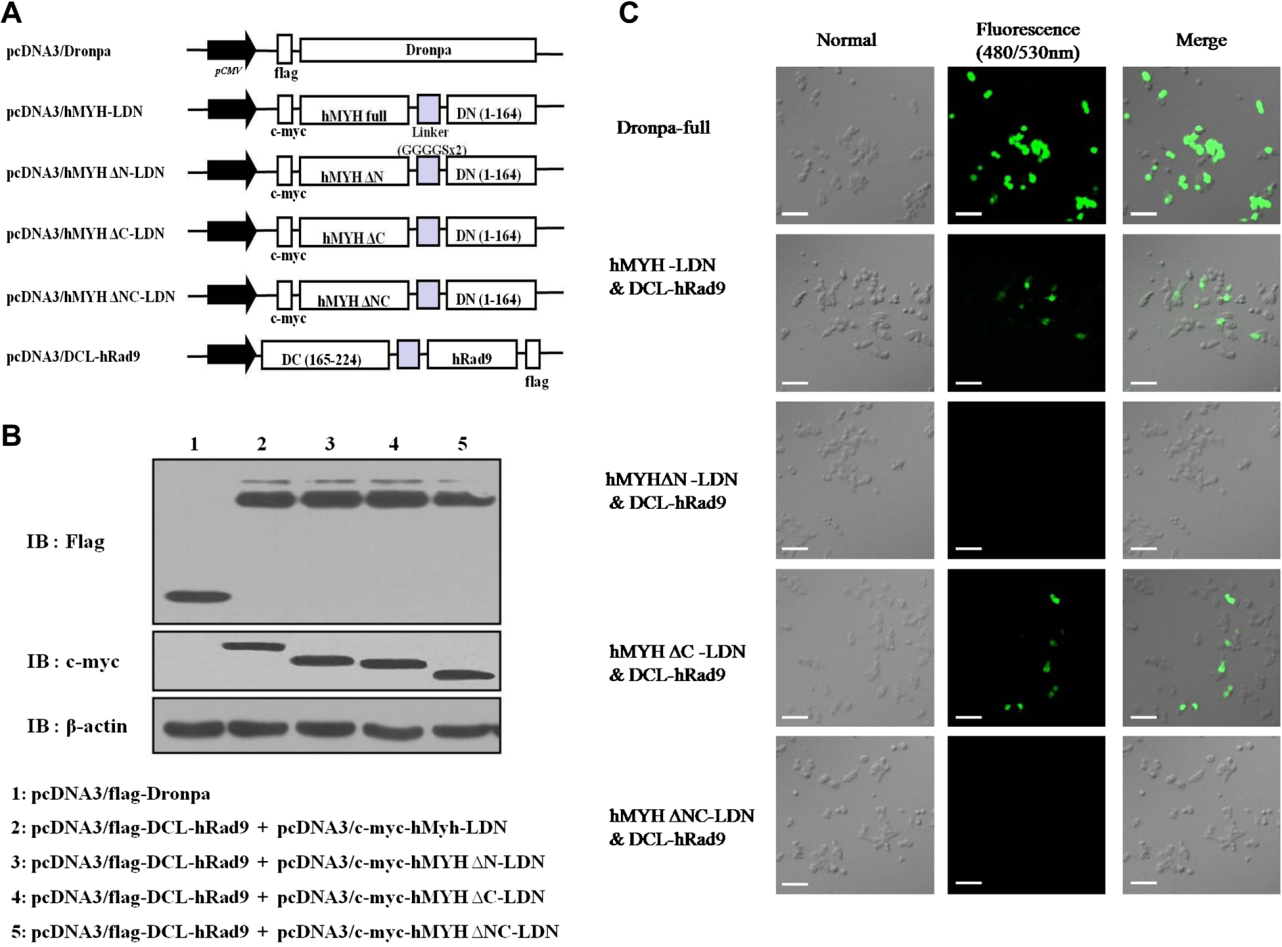
**hRad9 interacts with the N**-**terminal region of hMYH. (A)** Vector for the Dronpa-BiFC system used to identify the interacting region of hMYH. Expression vectors were created as previously described; however, the N-terminus of Dronpa (DN, 1–164) was C-terminally fused to either full-length, wild-type hMYH or mutant hMYH [ΔN, ΔC, ΔNC]. The C-terminal region of Dronpa (DC, 165–224) was N-terminally fused to hRad9. Dronpa and DCL-hRad9 were tagged with FLAG; hMYH-full-LDN and the deletion mutants were tagged with c-myc. **(B)** The expression of each protein in transfected HEK293 cells was detected by immunoblotting with anti-FLAG and anti-c-myc antibodies. **(C)** The Dronpa-BiFC system demonstrated that the N-terminal region of hMYH is important for the interaction between hMYH and hRad9. HEK293 cells were seeded on a cover glass-bottom dish at a density of 1 × 10^5^ cells per well. Cells were transfected with plasmids encoding full-length hMYH-LDN or a deletion mutant (hMYH-ΔN-LDN, hMYH-ΔC-LDN, or hMYH-ΔNC-LDN) and DCL-Rad9, as indicated to the left of the figure and incubated for 24 h. Fluorescence was assessed using a confocal fluorescence microscope with 488-nm excitation and 530-nm emission filters. Scale bar, 50 μm.

The N-terminal region of hMYH contains sites for RPA binding (amino acids 1–32) and function in substrate specificity, and the C-terminal region is important for glycosylase activity [19]. We created hMYH mutants with N-, C-, or N- and C-terminal deletions [ΔN (amino acids 75–547), ΔC (amino acids 1–487), and ΔNC (amino acids 75–487), respectively] to determine the functional impact of the hMYH and hRad9 interaction on substrate specificity and glycosylase activity. We successfully used the Dronpa-BiFC system developed in our lab to identify the region of hMYH that interacts with hRad9 and to visualize the interaction between hMYH-hHus1. The optimum fragments for Dronpa cleavage were selected according to a structural analysis of GFP and mRFP1. BiFC vectors for visualization of the interaction between hRad9 and hMYH were constructed as described in the Methods section [16]. The Dronpa C-terminus was fused to hRad9, and the Dronpa N-terminus was fused to either full-length hMYH (pcDNA3-c-myc/hMYH-LDN) or hMYH deletion mutants (pcDNA3-c-myc/hMYH∆N-LDN, pcDNA3-c-myc/hMYH-∆C-LDN, pcDNA3-c-myc/hMYH-∆NC-LDN). The vector used for the Dronpa-BiFC system is shown in Figure 2A. The transient expression of transfected proteins in cells was confirmed by immunoblotting (Figure 2B).HEK293 cells were transfected with different vector sets of plasmids (Dronpa-full, hMYH-LDN/DCL-hRad9, hMYH-∆N-LDN/DCL-hRad9, hMYH-∆C-LDN/DCL-hRad9, and hMYH-∆NC-LDN/DCL-hRad9). Cells were incubated for 24 h, and Dronpa fluorescence was visualized by fluorescence microscopy. Dronpa fluorescence was observed in cells transfected with native Dronpa and in cells co-transfected with hMYH-LDN/DCL-hRad9 and hMYH-∆C-LDN/DCL-hRad9, but no fluorescence was observed in cells transfected with hMYH-∆N-LDN/DCL-hRad9 and hMYH-∆NC-LDN/DCL-hRad9 (Figure 2C). These results showed that hRad9 interacts with the N-terminal region of hMYH. The Dronpa fluorescence observed in cells transfected with DCL-hRad9 and hMYH-LDN or hMYH-∆C-LDN was due to reconstitution of functional Dronpa facilitated by the interaction between hRad9 and hMYH.

**Figure 3:**
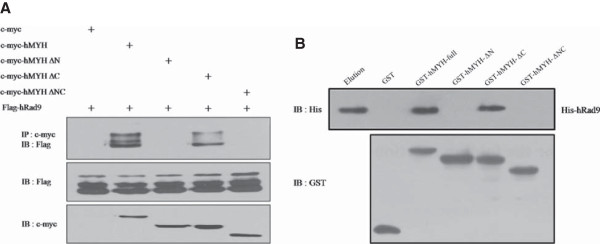
**hMYH physically interacts with hRad9. (A)** HEK293 cells were transfected with plasmids encoding c-myc, c-myc-hMYH-full, or a c-myc-tagged hMYH deletion mutants (−ΔN, −ΔC, or -ΔNC) and FLAG-tagged hRad9, as indicated at the top of the figure. Co-IP was performed with anti-c-myc antibody, and immunoprecipitates were immunoblotted with anti-FLAG and anti-c-myc antibodies. **(B)** hRad9 directly interacts with the N-terminus of hMYH. A GST pull-down assay was performed using GST, GST-hMYH-full, or a GST-hMYH deletion mutants (ΔN, ΔC, or ΔNC). Purified His-hRad9 was incubated with GST, GST-hMYH-full, or a GST-hMYH deletion mutants immobilized on glutathione beads. Bound proteins were separated by SDS-PAGE and detected with immunoblotting using anti-GST and anti-His antibodies. Lane 1: purified His-hRad9, lanes 2–6: pull-downs of His-hRad9 with GST (lane 2), GST-hMYH-full (lane 3), GST-hMYH-ΔN (lane 4), GST-hMYH-ΔC (lane 5), and GST-hMYH-ΔNC (lane 6).

Photoswitching activity used to identify Dronpa protein [16]. To evaluate the photoswitching activity of native or complemented Dronpa fragments, HEK293 cells were co-transfected with vectors expressing Dronpa or hMYH-LDN and DCL-hRad9. The cells were incubated for 24 h and analyzed by confocal fluorescence microscopy. After visualization, cells exhibiting Dronpa fluorescence were irradiated at 488 nm for 2 min to induce photobleaching and then photoactivated at 430 nm for 30 s. The similar results were obtained with native and complemented Dronpa fragments (data not shown).To confirm this interaction, we transfected different sets of vectors into HEK293 cells (c-myc, c-myc-hMYH-full, c-myc-hMYH-ΔN, c-myc-hMYH-ΔC, or c-myc-hMYH-ΔNC and FLAG-hRad9). After incubation for 24 h, cell lysates were extracted and immunoprecipitated with anti-c-myc antibody (Figure 3A). Immunoblot analysis was performed with anti-c-myc or anti-FLAG antibody. In agreement with Figure 2C, FLAG-hRad9 precipitated with c-myc-hMYH-full and c-myc-hMYH-ΔC (Figure 3A upper panel). To further demonstrate the direct interaction between hRad9 and hMYH, we performed a GST pull-down assay, as previously described. As same with IP results, Purified His-hRad9 was pulled down by GST-hMYH-full and GST-hMYH-ΔC, but not by GST alone, GST-hMYH-ΔN, and GST-hMYH-ΔNC (Figure 3B). These results indicate that the N-terminal region of hMYH facilitates the interaction between hMYH and hRad9.

### HU treatment increases the expression of hRad9 and hMYH, leading to cell cycle arrest

**Figure 4:**
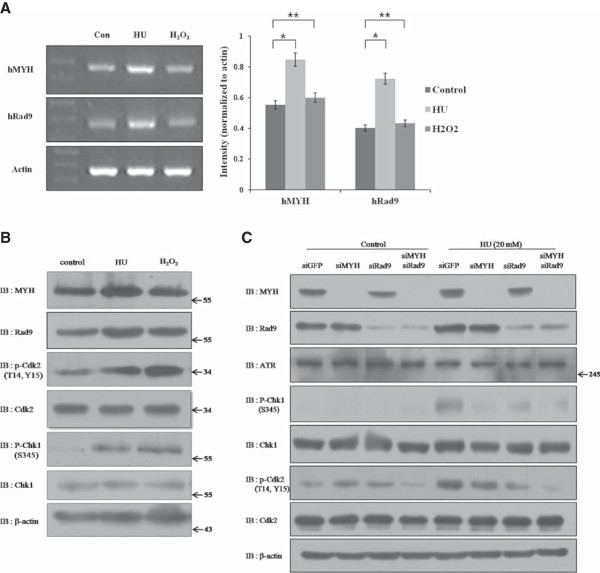
**HU induces hMYH and hRad9 expression. (A)** Reverse transcription-polymerase chain reaction (RT-PCR) analysis of hMYH and hRad9 mRNA expression. Total RNA was extracted from untreated or treated (HU or H_2_O_2_) cells and used for RT-PCR. The levels of hMYH and hRad9 mRNA were determined and normalized to β-actin levels (unpaired t-test; p < 0.05; *significant; **not significant) **(B)** hRad9 and hMYH expression in HEK293 cells increased after HU treatment, leading to cell cycle arrest at the G1/S phase. Total cell lysates from untreated, HU-treated, or H_2_O_2_-treated cells were used for immunoblotting to measure the expression of hMYH, hRad9, ATR, p-Chk1 (S345), Chk1, p-Cdk2 (T14, Y15), and Cdk2 proteins. **(C)** hRad9 and hMYH knockdown reduced the phosphorylation of Chk1 and Cdk2 in HU-treated cells. HEK293 cells were transfected with siRNA for GFP, hMYH, hRad9, or hMYH and hRad9 as indicated in the figure. Transfected cells were treated with 20 mM HU for 1 h, and then allowed to recover in fresh medium for 2 h. Total cell lysates were used for immunoblotting analysis with anti-hMYH, anti-hRad9, anti-ATR, anti-p-Chk1 (S345), anti-Chk1, anti-p-Cdk2 (T14, Y15), anti-Cdk2, and anti-actin antibodies.

We examined the physiological effects of the hRad9 and hMYH interaction by exposing cells to damaging agents (HU). hMYH and hRad9 mRNA levels were examined by RT-PCR. After normalization to actin, hMYH intensity was higher in HU-treated samples (0.85), compared to the intensity in untreated (0.55) and H_2_O_2_-treated (0.60) samples. The same result was observed for hRad9, which increased after HU treatment (0.72 vs. 0.40 in untreated and 0.43 in H_2_O_2_-treated). Thus, we noted a marked increase in hRad9 and hMYH mRNA levels after treatment with HU, but not after H_2_O_2_ treatment (Figure 4A). Moreover, hMYH and hRad9 protein expression also increased in HU-treated cells, but not in H_2_O_2_-treated cells.

HU arrests cell cycle progression by inhibiting ribonucleotide diphosphate reductase. p-Chk1, an indicator of DNA damage, was detected in both HU- and H_2_O_2_-treated cells (Figure 4B). p-Chk1 induces cell cycle arrest. Consistently, the phosphorylated, inactive form of Cdk2 (p-Cdk2, T14, Y15), which does not promote cell cycle progression, was higher in HU-treated cells.To further study the functional impact of the interaction between hRad9 and hMYH in HU-treated cells, we knockdown the expression of hMYH and/or hRad9 using siRNA. siGFP-transfected cells were used as a control. After transfection, cells were treated with HU, and protein expression was analyzed by immunoblotting. In siGFP-transfected cells, p-Chk1 (S345) and p-Cdk2 (T14, Y15) were detected, indicating that HU treatment induced cell cycle arrest (Figure 4C). p-Chk1 and p-Cdk2 levels were lower in HU-treated cells transfected with siRad9 and/or siMYH (Figure 4C). The reduction in p-Cdk2 was more pronounced in cells in which both hMYH and hRad9 were knocked down.

### The interaction and co-localization of hMYH and hRad9 in nuclear foci after exposure to genotoxic stress

**Figure 5:**
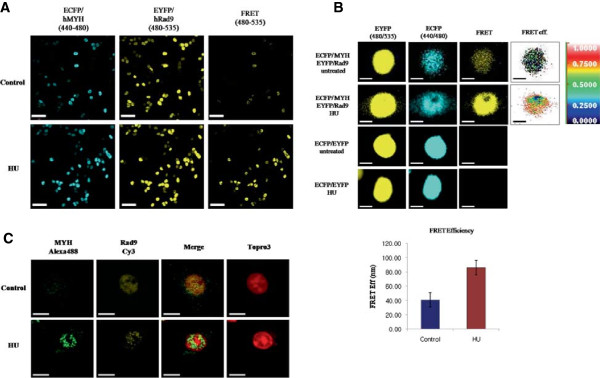
**DNA damage** (**HU**) **promotes the interaction between hRad9 and hMYH. (A)** Fluorescence resonance energy transfer (FRET) was used to analyze the interaction between over-expressed hRad9 and hMYH after DNA damage. Cells transfected with ECFP and EYFP or ECFP/hMYH and EYFP/hRad9 were treated with or without HU. ECFP, EYFP, and FRET fluorescence was observed with a fluorescence microscope at 440/480 nm (ECFP), 480/535 nm (EYFP), and 440/535 nm (FRET). Scale bar, 50 μm. **(B)** FRET efficiency was used to quantify the interaction between hMYH and hRad9 after DNA damage. HEK293 cells were transfected with ECFP and EYFP or ECFP/hRad9 and EYFP/hMYH as indicated to the left of the figure. Cells were treated with or without HU. ECFP, EYFP, and FRET fluorescence were measured at the indicated wavelengths. FRET efficiency was quantified in five control cells and five HU-treated cells. Standard error is shown. Scale bar, 10 μm. **(C)** Immunofluorescence of endogenous hMYH and hRad9. Cells were treated with or without 20 mM HU for 1 h and allowed to recover for 2 h. Cells were stained with antibodies against hMYH (FPG 456, green), hRad9 (FPR 552, yellow), and To-pro®-3 (nuclei, red). Scale bar, 10 μm.

The 9-1-1 complex and hMYH participate in DNA damage repair [2,4]. The interaction between hRad9 and hMYH after HU treatment was examined by FRET analysis. HEK293 cells were transfected with ECFP/hMYH and EYFP/hRad9, individually or together. Using confocal fluorescence microscopy, we detected ECFP and EYFP fluorescence in all transfected cells. FRET fluorescence was only observed in cells co-transfected with ECFP/hMYH and EYFP/hRad9. Moreover, higher intensity FRET fluorescence was seen in cells treated with HU, suggesting that HU treatment enhances the interaction between hRad9 and hMYH (Figure 5A).The interaction between hRad9 and hMYH exhibited a relatively high FRET intensity (Figure 5B). Given this result, we determined whether endogenous hMYH and hRad9 localized to the same nuclear foci after HU treatment. In immunofluorescence analysis, hMYH and hRad9 staining in the nucleus of untreated cells was faint (Figure 5C). However, in HU-treated cells, hMYH and hRad9 formed discrete nuclear foci, and a significant fraction of the hMYH nuclear foci co-localized with hRad9, indicating that hMYH and the 9-1-1 complex translocated to the same lesions after DNA damage.

## Conclusions

In this study, we showed that hRad9 interacts with hMYH by co-IP of over-expressed c-myc-hMYH and FLAG-hRad9 (Figure 1A) and co-IP of endogenous hMYH and hRad9 (Figure 1B). We confirmed the interaction in GST pull-down assays using GST-hMYH and His-hRad9 (Figure 1D). Moreover, we observed that interaction between hMYH and hRad9 was independent with DNA (Additional file 1). The results showed that the proteins interact directly. We also observed that hMYH interacts with hHus1 [16], and hRad1 (data not shown). In addition, the Dronpa-BiFC system, used previously to visualize the intercomplex interactions between hHus1 and hRad1 and between hMYH and hHus1 [16], was used to show that hRad9 interacts with hMYH (Figure 2C). Our results differ from those of a previous study by Shi *et al.*, in which hMYH was not detected in GST pull-down assays using GST-hRad9 [18]. Shi *et al.* investigated whether GST-hHus1, GST-hRad1, and GST-hRad9 pulled down His-hMYH. Their results showed that His-hMYH interacted directly with hHus1 and hRad1, but not with hRad9. They also found that GST-SpMYH pulled down the 9-1-1 complex. We suggest that the differences between their results and ours may be due to the different protein constructs used or the weak binding of hRad9 to hMYH [18,20,21].

In our lab, we found that disruption of hMYH and the 9-1-1 complex sensitizes cells to hydroxyurea (HU) and ultraviolet (UV) irradiation [22]. It is already known that 9-1-1 complex function in the early detection of DNA damage and assists other protein to bind to the site of damage [6-9]. On the other hand, hMYH function in creating AP-site in the early BER pathway [2]. Moreover, knockdown of hMYH and hRad9 induced the decrease of phosphorylation in the Chk1 and Cdk2, an upstream in the DNA damage repair pathway (Figure 4C). Therefore, we suggest that the interaction between hMYH and 9-1-1 occurs early in the DNA damage response and functions as an adaptor for other proteins at lesion sites to activate checkpoint control.

The hRad9 and hMYH interaction was studied by fluorescence resonance energy transfer (FRET). FRET occurs upon energy transfer from a donor molecule (ECFP) to an acceptor molecule (EYFP) [23]. Cells that over-expressed ECFP/hMYH and EYFP/hRad9 were treated with HU. FRET increased significantly in cells treated with HU (Figure 5A).

We also analyzed the interacting region of hMYH using the Dronpa-BiFC system. hMYH is significantly larger than the bacterial protein and comprises the entire MutY sequence plus extended N- and C-terminal flanking domains [24]. These 50–60 amino acids terminal domains are involved in subcellular targeting and interactions with other proteins. RPA and PCNA binding motifs, for example, map to the N- and C-terminus, respectively. These interactions suggest coupling of hMYH to the DNA replication machinery [2,24]. The Dronpa-BiFC system showed that hRad9 interacts with the N-terminal region of hMYH (Figure 2C). Subsequently, co-IP and GST pull-down assays showed that hRad9 interacts directly with the N-terminal region of hMYH (Figure 3A,B).

hMYH and hRad9 mRNA and protein levels increased after HU treatment, but not after H_2_O_2_ treatment (Figure 4A,B). HU inhibits ribonucleotide diphosphate reductase, thereby blocking DNA synthesis and repair [25]. When DNA is damaged, cell cycle progression arrests or slows to allow time for DNA repair [26]. The cell cycle checkpoint proteins Rad9-Rad1-Hus1 complex play important roles in both cell cycle checkpoint control and DNA repair [26]. Deletion of each gene encoding the three proteins in the fission yeast *S. pombe* inactivates S/M, intra-S, and G_2_/M checkpoint controls. Endogenous hMYH in HEK293 cells increased during S phase and decreased in M phase [22]. However, a basal level of hMYH was maintained during M phase. Disruption of hMYH levels reduced the amount of Chk1 activated by HU. These results indicate that, although mainly induced from late G1 to S phase, Chk1 activation can also be induced by DNA damage during G2/M phase and attenuated by hMYH disruption [22].

Chk1 on chromatin undergoes ATR-dependent phosphorylation in response to DNA damage. Phosphorylation appears to disrupt intramolecular interactions, leading to an open conformation of Chk1 and to checkpoint activation [27]. CDKs (Cyclin-dependent kinases) are heterodimeric serine/threonine protein kinases that control cell cycle progression. Among them, the Cdk1-cyclin B complex controls cell cycle progression in G2/M phase, and Cdk2-cyclin E/A complexes function in the G1/S and S/G2 transitions [28]. Cell cycle entry into mitosis is regulated by Cdk1 activation, which is controlled by cyclin binding and phosphorylation at T161. On the other hand, Cdk2 is activated during the progression of mitosis by dephosphorylation at T14 and Y15. Thus, phosphorylation of Cdk2 (T14, Y15) is indicative of cell cycle arrest [29].In cells depleted of hMYH and hRad9 by siRNA knockdown, p-Chk1 and p-Cdk2 expression levels decreased (Figure 4C). The decrease in p-Chk1 in single and double knockdowns indicates that hMYH and hRad9 are defective in promoting ATR activity. Therefore, we can conclude that hMYH and hRad9 interaction are important for the DNA damage response.

We suggest that hMYH and hRad9 interact early in the process of cell cycle arrest. Further studies to elucidate the mechanism that regulates interactions between hMYH and the 9-1-1 complex in response to different types of DNA damage are required.

## Methods

### Cell line and treatments

Human embryonic kidney (HEK293) cells were grown in Dulbecco’s modified Eagle’s Medium (DMEM; Welgene, Daegu, Korea) containing 10% fetal bovine serum (FBS; JR Scientific, Woodland, CA) and 1% penicillin-streptomycin solution (Welgene) at 37°C in a 5% CO_2_ incubator. Cells were seeded at 1 × 10^5^cells/ml then incubated overnight before transfection or treatment with damage reagent (20 mM HU for 1 h or 5 mM H_2_O_2_ for 40 min).

### Transient expression in HEK293 cells

Cells were transiently transfected using Lipofectamine™ 2000 reagent (Invitrogen, Carlsbad, CA) according to the manufacturer’s protocol and incubated 24 h. Cells were lysed in lysis buffer [50 mM Tris–HCl (pH 8.0), 100 mM NaCl, 5 mM EDTA, 1% Nonidet P-40, 10 g/ml PMSF, and protease inhibitor cocktail (Sigma, St. Louis, MO)] for 40 min at 4°C. Lysed cells were centrifuged for 20 min, and supernatants were collected for western blotting.

### Immunoprecipitation

Total cell lysates were incubated with anti-c-myc (Santa Cruz Biotechnology, CA, USA) or anti-FLAG antibody (Sigma) for 2 h and then with A/G PLUS-Agarose beads (Santa Cruz Biotechnology) overnight. Protein-bead complexes were precipitated by centrifugation, washed with phosphate-buffered saline (PBS; Sigma), and mixed with 2× SDS-PAGE loading buffer. The samples were then separated by SDS-PAGE and analyzed by immunoblotting. Endogenous proteins were co-immunoprecipitated using hMYH antibody (Abnova, Taipei, Taiwan) and the ImmunoCruz™ IP/WB Optima B System (Santa Cruz Biotechnology) according to the manufacturer’s protocol. Immunoblot analysis was conducted using mouse anti-MYH, anti-Rad9 (Novus Biologicals, Littleton, CO), and anti-Hus1 (Santa Cruz Biotechnology) antibodies.

### GST pull-down assay

A GST gene fusion system (Amersham Biosciences, Uppsala, Sweden) was used to generate GST-tagged hMYH wild type and mutants (ΔN, ΔC, and ΔNC). For GST pull-down assays, fusion proteins were adsorbed to glutathione-Sepharose 4B beads. His-purified hRad9 protein was then incubated with GST or GST fusion protein in binding buffer [50 mM Tris–HCl (pH 7.5), 150 mM NaCl, 1 mM EDTA, 0.3 mM DTT, 0.1% NP-40, and protease inhibitor cocktail], and proteins were incubated for 3 h at 4°C. The beads were washed, and bound proteins were separated by SDS-PAGE analysis and assessed by immunoblotting.

### Western blotting

Proteins were fractionated by an 8% or 10% SDS-PAGE and transferred to a polyvinylidene fluoride (PVDF) membrane (PALL Corporation, New York, NY). Membranes were blocked with 3% skim milk and washed with TBS-Tween 20. Membranes were then incubated with antibodies against hMYH, hRad9, hHus1, c-myc, FLAG, and ATR; p-Chk1 (S345); Chk1; p-Cdk2 (T14, Y15); Cdk2 (Santa Cruz Biotechnology) then incubated with appropriate horseradish peroxidase-conjugated secondary antibodies (Santa Cruz Biotechnology). Protein bands were detected using ECL Pico western blotting detection reagents (Pierce, Rockford, IL).

### The Dronpa-BiFC system

The intact Dronpa molecule and non-fluorescent fragments were fused to hMYH or hRad9 and inserted into the pcDNA3-c-myc vector. The construction of a Dronpa N-terminal and C-terminal fragment fused to a flexible linker as described by Lee *et al.*[16]. Gene fragments corresponding to the coding regions of hMYH and hRad9 (accession numbers: hMYH, NM_012222; hRad9, NM_004584) were amplified by PCR using a HotStarTaq kit (Qiagen, Venlo, Netherlands).

### hMYH full-length (amino acids 1–547), ΔN (amino acids 75–547), ΔC (amino acids 1–487), and ΔNC (amino acids 75–487) constructs

Specific primers were used to amplify the hMYH ΔN, ΔC, and ΔNC regions of the hMYH gene. The PCR products were cleaved and ligated into *Hind*III-*Xho*I-digested pCMV-tag3A-c-myc or pGEX-4 T1 (GE Healthcare, Princeton, NJ) vectors. All constructs were confirmed by restriction enzyme mapping and DNA sequence analysis.

### ECFP/hMYH-full, −ΔN, −ΔC, and -ΔNC and EYFP/hRad9

Gene fragments corresponding to the complete coding region of hMYH-full, −ΔN, −ΔC, −ΔNC, and the complete coding region of hRad9 were amplified by PCR and subcloned into *Hind*III-*Xba*I-digested ECFP vector and *EcoR*1-*Hind*III-digested EYFP vector (Clontech, Mountain View, CA).

### Dronpa fluorescence analysis

HEK293 cells were seeded on a cover glass-bottom dish (SPL, Pocheon, South Korea), and co-transfected with the indicated vectors. Cells were fixed with 4% paraformaldehyde. After washes with PBS, Dronpa fluorescence was visualized under a confocal fluorescence microscope (Olympus FV-1000; software, Olympus FluoView; Olympus, Center Valley, PA) using 488-nm excitation and 530-nm emission filters. After visualization of Dronpa fluorescence, the cells were irradiated with a 488-nm laser for 2 min to induce photobleaching, followed by irradiation with a 430-nm excitation laser for 30 s. Photoswitching activity was monitored for several cycles.

### siRNA transfection

The siRNA sequences 5′-GGGCACAAGCUGGAGUACAACUACA-3′ (Santa Cruz Biotechnology) and 5′-CACACAGUUGGAUAAACAU-3′ (Bioneer, Daejeon, Korea) were used to target GFP and Hus1, respectively. HEK293 cells were transfected with each siRNA and incubated for 24 h. GFP was used as a negative control.

### Reverse transcription-PCR (RT-PCR)

HEK293 cells were treated with H_2_O_2_ or HU as described previously. RNA was extracted using a RiboEx total RNA isolation kit (GeneAll Biotechnology, Seoul, Korea) according to the manufacturer’s protocol. RNA was reverse-transcribed for RT-PCR using Omniscript reverse transcriptase (Qiagen) by following the manufacturer’s instructions. RT-PCR was carried out for 1.5 h at 37°C with primers specific for hMYH and. β-actin was used as a control. DNA was amplified for 30 cycles, and analyzed by agarose gel electrophoresis.

### Fluorescence resonance energy transfer (FRET)

Cells (0.7 × 10^5^) were seeded on cover glass-bottom dish and incubated overnight. Cells were transfected with ECFP/EYFP, ECFP/hMYH, or EYFP/hRad9 or co-transfected with ECFP-EYFP or ECFP/hMYH-EYFP/hRad9. After 24 h of incubation, cells were treated with HU or untreated as previously described. Cells were fixed with 4% paraformaldehyde. After incubation, cells were washed with PBS. FRET fluorescence were observed with a confocal fluorescence microscope (Olympus FV-1000; software, Olympus FluoView Ver. 2.0c).

### Immunofluorescence

Cells were grown on a cover glass-bottom dish (1 × 10^5^) and then treated with HU as previously described. Immunofluorescence was performed as described by Shi *et al.*[18]. Cells were blocked with 15% FBS then reacted with hMYH antibody or hRad9 antibody. Cells were washed and incubated with anti-mouse FPG 456 (BioActs, Incheon, Korea) or anti-rabbit FPR 552 (BioActs) antibody. The cells were washed with PBS, and nuclear DNA was counterstained with To-pro®-3 (Invitrogen). Images were captured with a confocal fluorescence microscope (Olympus FV-1000).

## Abbreviations

hMYH: Human MutY glycosylase homolog; BiFC: Bimolecular fluorescence complementation; FRET: Fluorescence resonance energy transfer; HU: Hydroxyurea; siRNA: Small interfering RNA; GFP: Green fluorescent protein; YFP: Yellow fluorescent protein; CFP: Cyan fluorescent protein; His: Poly-histidine; GST: Glutatione S-transferase; ATR: Ataxia telangiectasia and Rad3-related protein; Chk1: Checkpoint kinase 1.

## Competing interests

The authors have declared that no competing interests exist.

## Authors' contributions

LA, SH, and SHH were major contributors in this study. LA, SH conceived the idea and designed experiment. JHC, JWP and JP contributed to idea. LA, SHH, and AHVT performed experiment. LA, SH, SHH and YSH wrote the manuscript. All authors read and approved the final manuscript.

## Supplementary Material

Additional file 1 **hMYH interacts with hRad9 in DNA-independent manner.** HEK293 cell were treated or untreated Dnase for 20 min, in 37°C. Cells were lyased and conducted IP essay using anti-hRad9 antibody. Click here for file
