# Supplementary material for: Visualization of the physical and functional interaction between hMYH and hRad9 by Dronpa bimolecular fluorescence complementation
Source: BMC Mol Biol. 2014 Aug 15;15:17. doi: 10.1186/1471-2199-15-17 (PMC4151078; doi:10.1186/1471-2199-15-17)
Supplement: Additional file 1 — hMYH interacts with hRad9 in DNA-independent manner. HEK293 cell were treated or untreated Dnase for 20 min, in 37°C. Cells were lyased and conducted IP essay using anti-hRad9 antibody. [file 1471-2199-15-17-S1.pptx]

## Slide 1
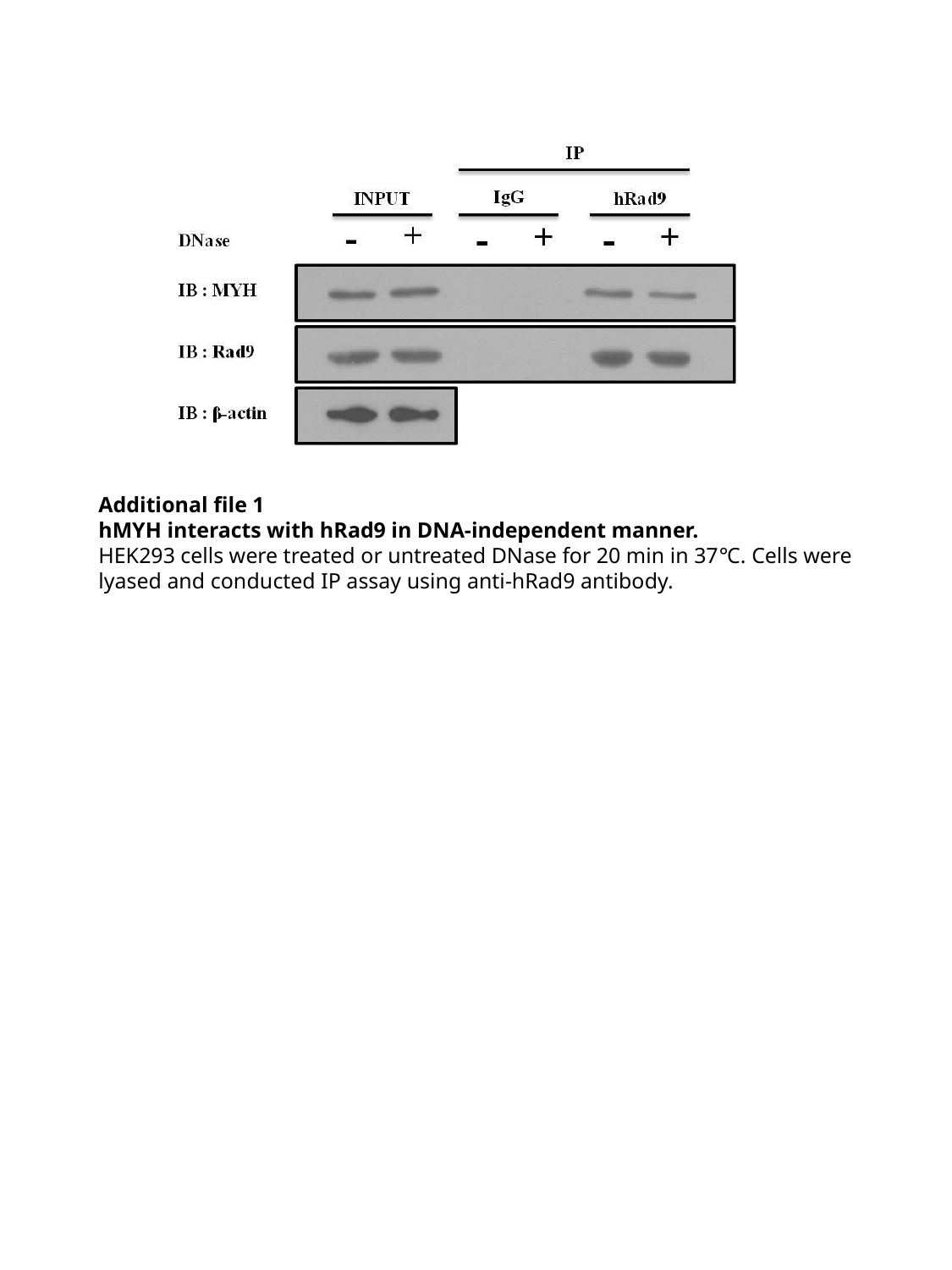

Additional file 1
hMYH interacts with hRad9 in DNA-independent manner.
HEK293 cells were treated or untreated DNase for 20 min in 37℃. Cells were lyased and conducted IP assay using anti-hRad9 antibody.
